# *DNMT3A* mutations in Chinese childhood acute myeloid leukemia

**DOI:** 10.1097/MD.0000000000007620

**Published:** 2017-08-04

**Authors:** Weijing Li, Lei Cui, Chao Gao, Shuguang Liu, Xiaoxi Zhao, Ruidong Zhang, Huyong Zheng, Minyuan Wu, Zhigang Li

**Affiliations:** a Hematology & Oncology Laboratory, Beijing Pediatric Research Institute; b Beijing Key Laboratory of Pediatric Hematology Oncology; c Key Laboratory of Major Diseases in Children; d National Key Discipline of Pediatrics, Ministry of Education; e Hematology & Oncology Center, Beijing Children's Hospital, Capital Medical University, National Center for Children's Health, Beijing, China.

**Keywords:** childhood acute myeloid leukemia, DNA methyltransferase 3A mutations, FLT3 internal tandem duplication, prognosis

## Abstract

**Background:**

DNA methyltransferase 3A (*DNMT3A*) mutations have been found in approximately 20% of adult acute myeloid leukemia (AML) patients and in 0% to 1.4% of children with AML, and the hotspots of mutations are mainly located in the catalytic methyltransferase domain, hereinto, mutation R882 accounts for 60%. Although the negative effect of DNMT3A^R882^ on treatment outcome is well known, the prognostic significance of other *DNMT3A* mutations in AML is still unclear. Here, we tried to determine the incidence and prognostic significance of *DNMT3A* mutations in a large cohort in Chinese childhood AML.

**Methods:**

We detected the mutations in *DNMT3A* exon 23 by polymerase chain reaction and direct sequencing in 342 children with AML (0–16 years old) from January 2005 to June 2013, treated on BCH-2003 AML protocol. The correlation of *DNMT3A* mutations with clinical characteristics, fusion genes, other molecular anomalies (FLT3 internal tandem duplication [*FLT3-ITD*], Nucleophosmin 1, C-*KIT* (KIT proto-oncogene receptor tyrosine kinase), and Wilms tumor 1 mutations), and treatment outcome were analyzed.

**Results:**

*DNMT3A* mutations were detected in 4 out of 342 (1.2%) patients. Two patients were *PML-RARA* positive and 1 patient was *FLT3-ITD* positive. The mutations in coding sequences included S892S, V912A, R885G, and Q886R. Furthermore, there was 1 intronic mutation (c.2739+55A>C) found in 1 patient. No association of *DNMT3A* mutations with common clinical features was found. Two patients with *DNMT3A* mutations died of relapse or complications during treatment. One patient gave up treatment due to remission induction failure in day 33. Only 1 patient achieved continuous complete remission.

**Conclusions:**

*DNMT3A* mutations were rare in Chinese children with AML including *PML-RARA* positive APL. The mutation positions were different from the hotspots reported in adult AML. *DNMT3A* mutations may have adverse impact on prognosis of children with AML.

## 1 Introduction

Acute myeloid leukemia (AML) accounts for 20% of childhood leukemias. Although the 5-year event-free survival (EFS) for children with newly diagnosed AML approaches 50% to 60%, relapsed or refractory AML remains the most challenging problems in treatment of this disease.^[1–4]^

Leukemia is a heterogeneous disease characterized by specific genetic alterations and related multiple epigenetic changes.^[5]^ DNA methyltransferase 3A (*DNMT3A*) mutations have been found in approximately 20% to 25% of adult AML patients and only 0% to 1.4% of childhood AML.^[5–20]^ The hotspots of mutations are mainly located in the catalytic methyltransferase domain, hereinto, mutation R882 accounts for 60%, which conferred significant adverse prognostic impact.^[5–19,21]^ However, the prognostic significance of other *DNMT3A* mutations in AML is to be determined. Here, we reported the incidence and prognostic significance of *DNMT3A* mutations in a large cohort of Chinese children with AML.

## 2 Materials and methods

### 2.1 Patients

A total of 342 children with AML, including 3 cases with acute myeloblastic leukemia, without maturation (M1), 142 cases with acute myeloblastic leukemia with granulocytic maturation (M2), 76 cases with acute promyelocytic leukemia (M3), 43 cases with acute myelomonocytic leukemia (M4) or together with bone marrow eosinophilia (M4Eo), 33 cases with acute monoblastic leukemia (M5), 11 cases with acute erythroid leukamia (M6), 29 cases with acute megakaryoblastic leukemia (M7), and 5 cases with mixed phenotype acute leukemia (MPAL), were enrolled in this study. The patients were diagnosed as AML from January 2005 to June 2013 at Beijing Children's Hospital. There were 208 boys and 134 girls, aged from 1 to 16 years with a median of 7 years. One hundred and ninety-seven patients were found positive for different types of fusion genes including *AML1-ETO*, *PML-RARA*, and *CBFβ-MYH11*, *MLL* rearrangements, *DEK-CAN* and *TLS-ERG* fusion. The patients were treated in accordance with the AML BCH-2003 Protocol. Informed consents were obtained from all the children's parents or legal guardians.

### 2.2 Nucleic acid extraction

Ficoll 400 (MD Pacific Technology Co., Ltd., Tianjin, China) was used to isolate bone marrow mononucleated cells. The cells were stored at −70 °C until use. We used the DNA Extraction Kit (U-gene Co., Ltd., Anhui, China) to extract genomic DNA. Trizol Reagent (Invitrogen, Carlsbad, CA, Promega, Madison, WI) was used to extract total RNA. Then the RNA was reverse transcribed into cDNA with moloney murine leukemia virus reverse transcriptase (Invitrogen; Promega).^[22]^

### 2.3 Detection of DNMT3A mutations

In this study, the mutations in exon 23 as well as adjacent intronic regions were focused, because most of *DNMT3A* mutations were concentrated in exon 23. The polymerase chain reaction (PCR) mixture was 50 μL, containing 100 ng of genomic DNA, 5 μL of 10 times buffer, 1.5 mM MgCl_2_, 0.2 mM dNTPs, 10 pmol of upstream and downstream primers, and 1 to 2 U of Taq DNA polymerase (Promega, USA). The primers’ sequences could be found in Table 1. The cycling condition was as follows: 5 minutes at 95 °C for predenaturation, 40 cycles of 30 seconds at 95 °C, 30 seconds at 55 °C, and 30 seconds at 72 °C, and 10 minutes at 72 °C for final extension. The PCR products were sent to Shanghai Sangon Biological Engineering Technology & Service Co., Ltd. and directly sequenced using AB PRISM 3730 Automated Sequencer. Four PCR products with unsatisfactory sequencing results were subcloned into *pEASY*^*TM*^-T5 Zero cloning vector. The recombinant plasmids were transformed into Trans 5α chemically competent cell (Transgene, Beijing, China). Five to 10 clones for each product were selected for plasmid sequencing after incubation at 37 °C for 15 hours.^[22]^

**Table 1:**
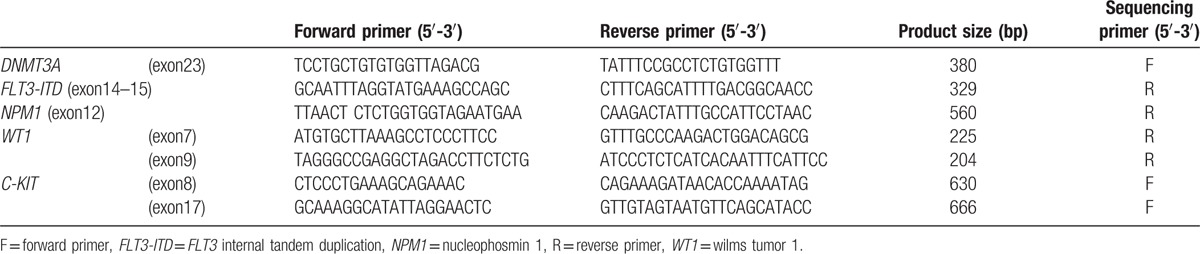
Primers for mutation detection in childhood AML.

### 2.4 Analysis and interpretation of DNMT3A mutations

Mutation Surveyor software (SoftGenetics, PA) was used to analyze the sequencing results for existence of mutations. Variant effect predictor (Ensembl, Sanger Institute, Cambridge, United Kingdom) online tool and mutation taster online tool were used to analyze the possible effect on protein function for every mutation of *DNMT3A*. The National Center for Biotechnology Information record NM_175629 was used as the reference sequence of *DNMT3A*.

### 2.5 Detection of the other genetic aberrations and mutations

Twenty-nine types of fusion genes resulting from chromosome translocations were detected in cDNA samples as described previously.^[23]^ The other common genetic mutations in AML were also detected in genomic DNA samples including Nucleophosmin 1 (*NPM1*), *C-KIT*, Wilms tumor 1 (*WT1*) mutation, and *FLT3* internal tandem duplication (*FLT3-ITD*), as described elsewhere.^[2–4,24–28]^ The primers sequences were presented in Table 1.

### 2.6 Statistical analysis

The difference in clinical characteristics between the 2 groups of patients with or without *DNMT3A* mutations was tested using Fisher exact test. SPSS 16.0 software (SPSS Inc., Chicago, IL) package was used to statistically analyze all of the data. *P* < .05 was appointed statistically significant.

## 3 Results

### 3.1 DNMT3A mutations in childhood AML

**Figure 1:**
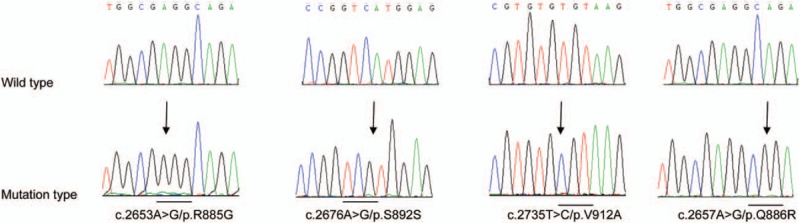
Four mutations in *DNMT3A* coding sequences detected in pediatric AML. Mutated nucleotides were indicated by arrows and changes of the codons were underlined. AML = adult acute myeloid leukaemia, DNMT3A = DNA methyltransferase 3A.

In total, 4 out of 342 (1.2%) patients with newly diagnosed AML were identified with *DNMT3A* mutations by direct sequencing and clone sequencing for exon 23. *PML-RARA* fusion gene was detected in 2 patients’ leukemic blasts. No other fusion gene was found in the other 2 patients. Three missense mutations and one synonymous mutation in exon 23 including V912A, R885G, Q886R, and S892S were detected in 4 patients respectively (Fig. 1 and Table 2). In patient 3, we also detected one mutation in intronic regions: c.2739+55A>C (Table 2). Only S892S has been reported previously,^[13]^ the other 4 were all novel variants (Table 3). However, the most common mutation R882 was not found in our series of cases. Of note, the *DNMT3A* mutations detected in diagnostic samples did not exist in corresponding complete remission (CR) samples. These findings indicated that these mutations were leukemia specific and were not germline.

**Table 2:**
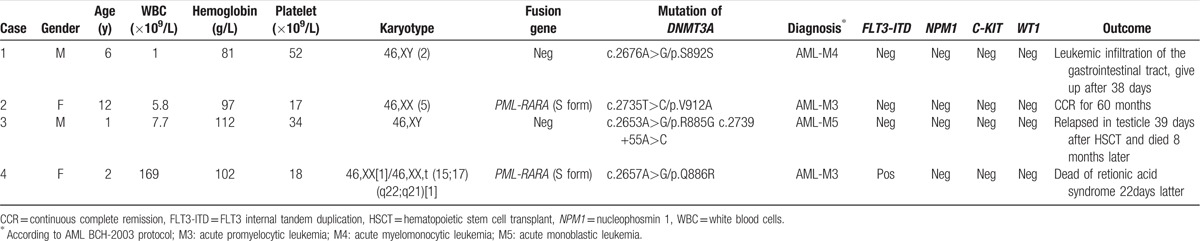
Clinicobiological characteristics and treatment outcome of childhood AML with *DNMT3A* mutations.

**Table 3:**
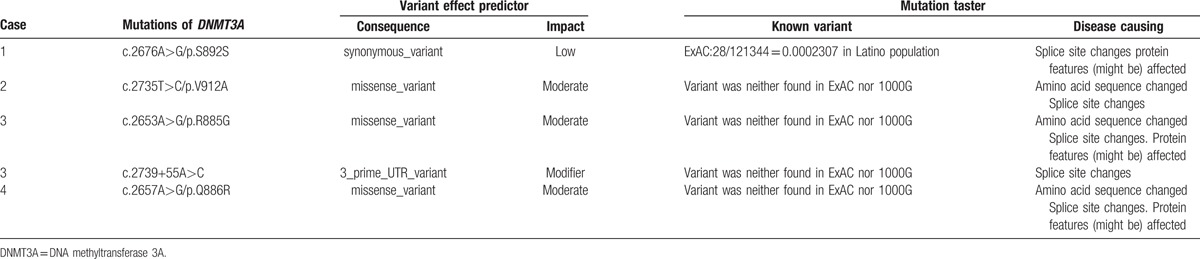
The interpretation of mutations of *DNMT3A*.

In order to assess the possible effect of the 5 mutations on protein structure and function, we analyzed the mutations with variant effect predictor and mutation taster. The 3 missense mutations, V912A, R885G, and Q886R, changed amino acid sequence as well as splice site, with moderate impacts. In addition, the latter 2 might affect protein features. Although S892S and c.2739+55A>C made no effect on amino acid sequence, these 2 mutations changed splice site. Interestingly, the former might also affect protein features according to analysis result of mutation taster (Table 3).

### 3.2 Correlation of DNMT3A mutations with clinical characteristics

We firstly analyzed the association of *DNMT3A* mutations with other 4 gene (*NPM1*, *C-KIT*, *WT1*, and *FLT3-ITD*) mutations respectively, but found no significant correlation with them (Fisher exact test, *P* > .05, Table 4). Furthermore, no correlation was found between the common clinical features including age, sex, diagnostic white blood cell count, leukemia subtype, karyotype, fusion genes, and *DNMT3A* mutations (Table 4).

**Table 4:**
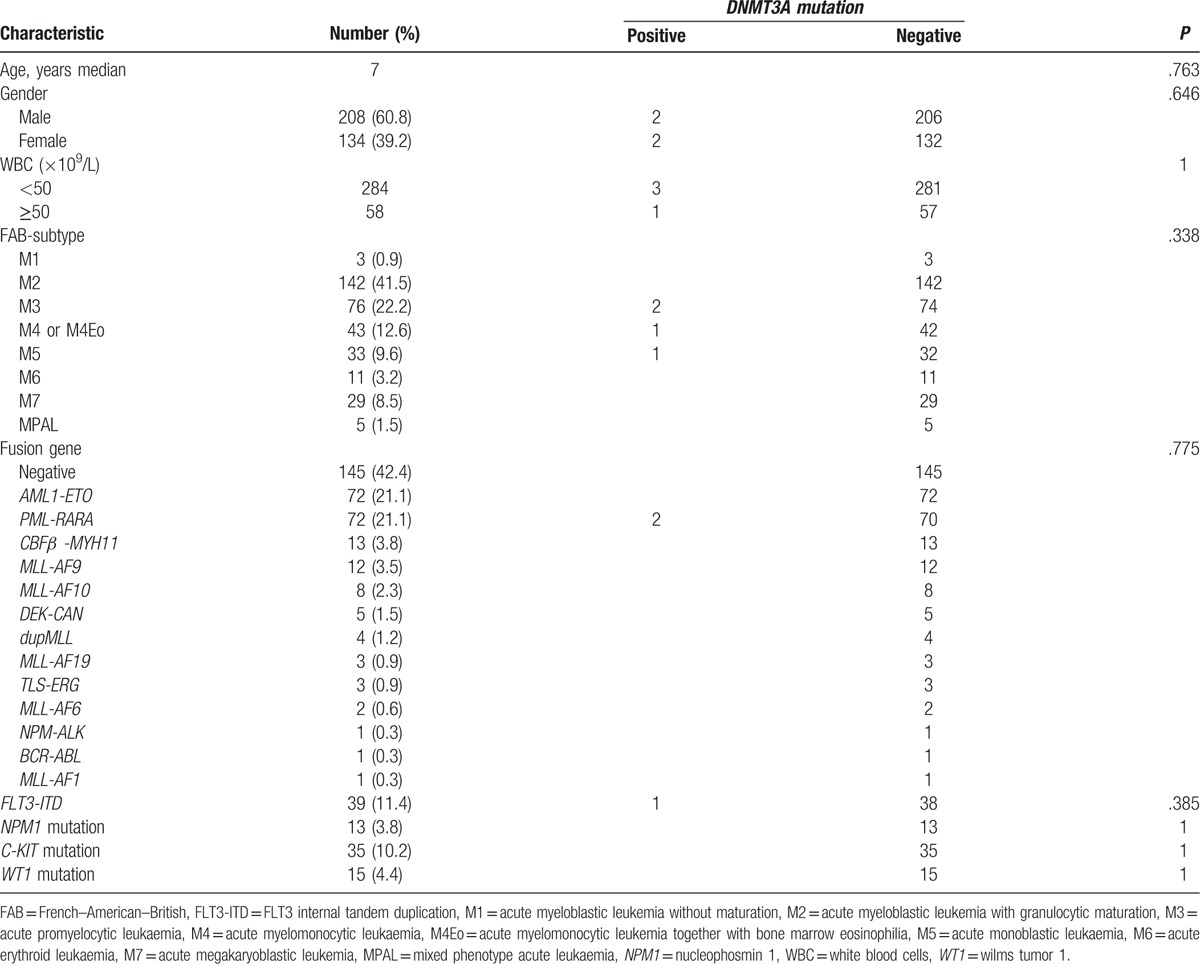
Association of *DNMT3A* mutation with common clinical characteristics.

### 3.3 Clinical outcome of patients’ with DNMT3A mutations

*DNMT3A* mutations were detected in 4 out of 342 (1.2%) patients. Of those with *DNMT3A* mutated, patient 1 (man, 6 years old, FAB M4) harboring a S892S mutation was diagnosed as leukemic infiltration of the gastrointestinal tract, and gave up treatment after 38 days due to remission induction failure at day 33. Patient 2 (woman, 12 years old, APL) harboring a V912A mutation and *PML-RARA* fusion gene achieved continuous complete remission for 60 months. Patient 3 (man, 1 year old, FAB M5) harboring a R885G mutation and 1 intronic mutations (c.2739+55A>C) was diagnosed as AML with testicular and CNS involvement. After 10 months, the patient undertook hematopoietic stem cell transplant but relapsed again in testicle after 39 days and died 8 months later. It was suggested that complex mutations of *DNMT3A* may associated with poor prognosis. It was noteworthy that 2 mutations (R885G and c.2739+55A>C) detected at diagnosis could not been found at testicular relapse in patient 3. Thus, it was possible that mutations in other genes might occur in the relapse clone. Patient 4 (woman, 2 years old, APL) harboring a Q886R mutation was found to be *PML-RARA* and *FLT3-ITD* positive, and died of retionic acid syndrome 22 days later (Table 2).

## 4 Discussion and conclusions

In former reports, *DNMT3A* mutation has been found in approximately 20% of adult AML patients and in 0% to 1.4% childhood AML.^[7,11–15,19]^ The hotspots of mutations are mainly located in the catalytic methyltransferase domain, hereinto, R882 mutation accounts for 60% in adult AML.^[13,17,19,29]^ Thus, in the present study, we focused on the mutations in exon 23 which encoded the catalytic methyltransferase domain of DNMT3A. We found that *DNMT3A* mutations can be found in 1.2% of Chinese children with AML. This incidence was in accordance with the previous results.^[5–20]^ However, the mutation positions were different from those reported in adult AML (Fig. 1 and Table 2): of the 5 mutations (S892S, V912A, R885G, Q886R, and c.2739+55A>C), only the synonymous mutation, S892S, has been reported in ExAC (28/121344, 0.02307% in Latino), the other 4 mutations were all reported for the first time to our knowledge (Table 3). These results indicated the distinctive features of *DNMT3A* mutations in childhood AML.

S892S mutation changed the splice site and might affect the protein features according to mutation taster tool (University of Berlin,Germany). However, Pt.1 with DNMT3A^[S892S]^ gave up treatment due to leukemic infiltration of the gastrointestinal tract in the early phase of treatment, we were not sure about the association of S892S with prognosis. V912A mutation was a fairly subtle amino acid change and did not affect the protein features (Table 3). As codon 912 was at the end of the protein (stop codon was at 913), it is difficult to determine if the V912A mutation would be detrimental to DNMT3A protein function, and whether it was associated with patient 2's continuous complete remission (CCR) for 60 months. Both R885G and Q886R mutations were located in the methyltransferase domain and very close to hotspot R882. Thus, we speculated that these 2 mutations may play a role similar to that of mutation at R882, which is associated with poor prognosis. Mutation c.2739+55A>C which was located at 3′UTR changed the splice site of DNMT3A. However, the exact impact on the protein function and the association of this mutation with the poor prognosis of Pt.3 needed to be clarified in further functional experiments.

There have been few reports of *DNMT3A* mutations in patients with *PML-RARA.* A study has figured out that APL patients were generally excluded from many studies because of existence of the unique fusion gene *PML-RARA* and the favorable prognosis in contrast to other subtypes. Moreover, *DNMT3A* gene mutation was almost never found in APL patients.^[6,21]^ However, in our study, *DNMT3A* mutations were found in 2 patients (Pt.2 and Pt.4) with APL and *PML-RARA* fusion gene, indicating the requirement of detection of *DNMT3A* mutations in childhood APL.

Now, more and more studies have shown that *DNMT3A* is a haploinsufficient tumor suppressor gene in myeloid leukemias, when cooperating mutations are present.^[30–32]^ In the absence of high-risk cytogenetics, *DNMT3A* mutation status has an adverse impact on outcome in the presence of *FLT3* and/or *NPM1* mutations.^[26]^ The R882H mutation associated with AML dominantly inhibits wild-type DNMT3A by blocking its ability to form active teramers.^[33]^*DNMT3A* loss drives enhancer hypomethylation in *FLT3-ITD*-associated leukemias.^[34]^ Coexistence of *DNMT3A* R882 mutation and *FLT3-ITD* was an extremely poor prognostic factor in patients with normal-karyotype AML after allogeneic hematopoietic cell transplantation.^[35]^ Notably in our study, the poor treatment outcome of Pt.4 in this study was consistent with the above results. Although both of Pt.2 and Pt.4 have *PML-RARA* fusion gene and mutated *DNMT3A*, the outcome of these 2 patients was quite different. Pt.4 (*PML-RARA,* DNMT3A^Q886R^, and *FLT3-ITD* positive) died at early phase of treatment, whereas Pt.2 (*PML-RARA* and DNMT3A^V912A^) had been in CCR for 60 months, suggesting that *FLT3-ITD* acts as a key role to accelerate the progress in Pt.4 with APL and DNMT3A^Q886R^. *DNMT3A* mutations alone may not induce AML, only acts as an initial lesion and requires an additional genetic event to increase susceptibility to leukemic development.

The clinical significance of adult AML with *DNMT3A* mutations seems to be age dependent.^[10,21]^*DNMT3A*-R882 mutation are associated with adverse prognosis in older patients (≥60), and non-R882-*DNMT3A* mutations are associated with adverse prognosis in younger patients (18< and <60). This finding may also be available and suitable in childhood AML. In this study, the median age of the 4 patients with non-R882 *DNMT3A* mutations was 7 years old. In the 3 patients younger than 7, 2 patients (Pt.3 and Pt.4) died of relapse or complications, the other patient gave up treatment because of remission induction failure in day 33. In contrast, the only 1 patient older than 7 (Pt. 2) were in CCR up to 60 months. The mechanisms behind this phenomenon were worth investigating.
